# Dietary supplementation with corn distillers’ dried grains with solubles improves egg quality in Haidong chickens by promoting beneficial gut microbiota and modulating serum biochemical profiles

**DOI:** 10.3389/fmicb.2025.1709407

**Published:** 2025-11-28

**Authors:** Gaoliang Bao, Hong Wei, Decang He, Dejun Wang, Baoan Ding, Junxia Zhang, Jiayu Tian, Pengshun Wang, Baolin Shen

**Affiliations:** 1 College of Agriculture and Animal Husbandry, Qinghai University, Xining, China; 2 Department of Animal Science and Veterinary Medicine, Qinghai University, Xining, China; 3 Animal Husbandry and Veterinary Station of Huzhu County of Qinghai Province, Huzhu, China

**Keywords:** DDGS, Haidong chicken, egg quality, gut microbiota, serum biochemical profiles

## Abstract

Gut microbial metabolism plays a critical role in modulating egg quality through its influence on host serum physiology. Recent evidence suggests that distillers’ dried grains with solubles (DDGS) exhibit prebiotic properties by promoting beneficial gut microbiota while suppressing pathogenic bacteria, thereby enhancing intestinal microbial homeostasis. This study investigated the impacts of dietary DDGS supplementation on egg quality, serum physiological-biochemical indices, and gut microbial communities in Haidong chickens. A total of 288 hens were randomly allocated to six dietary treatments containing 0, 2.5, 5.0, 7.5, 10.0, and 12.5% DDGS for 90 days, followed by the collection of eggs, serum samples, and intestinal contents for analysis. The results indicated that 12.5% DDGS supplementation significantly improved egg albumen height and egg weight (*p* < 0.05). Concurrently, serum analyses revealed enhanced immune indices, elevated protein metabolism markers, increased high density lipoprotein (HDL) and high density lipoprotein cholesterol (HDL-C) levels, and up-regulated anti-inflammatory cytokine IL-10. Conversely, the 12.5% DDGS group exhibited reduced lipid parameters, decreased liver function marker ALT, and down-regulated pro-inflammatory cytokines (*p* < 0.05). Gut microbiota profiling showed that the 12.5% DDGS group significantly increased the relative abundances of *Firmicutes* and *Lactobacillus* (*p* < 0.05). Moreover, *Lactobacillus pontis* and *Romboutsia ilealis* are associated with enhanced gut immunity and dietary nutrient metabolism, as well as host growth and developmental processes, contributing to the observed improvements in egg quality. These findings establish DDGS as a functional feed additive that improves poultry productivity through microbiota-mediated mechanisms, providing valuable insights for its application in sustainable animal husbandry practices.

## Introduction

The Haidong chicken, a distinctive indigenous breed native to the Haidong region of China, represents a valuable genetic resource for egg and meat poultry production. This breed exhibits remarkable adaptability to high-altitude environments, demonstrating exceptional tolerance to hypoxic conditions and cold climates characteristic of semi-agricultural and semi-pastoral regions (Ding et al., 2019). Its desirable traits include superior meat flavor, low fat deposition, high egg quality, disease resistance, and adaptability to free-range systems with coarse feed utilization. However, long-term semi-intensive and semi-extensive management systems have led to nutritional imbalances in rearing practices, particularly manifesting as retarded growth during the juvenile phase (Ding et al., 2019). The global livestock industry currently faces critical challenges regarding sustainable protein supply, with soybean meal shortages and price volatility significantly impacting feed production costs (Swiatkiewicz et al., 2016). These constraints necessitate an urgent exploration of cost-effective alternatives to protein supplementation for specialized poultry breeds such as the Haidong chicken. Distillers’ dried grains with solubles (DDGS), a co-product of corn ethanol production, emerges as a promising unconventional protein source. Containing 26–32% crude protein along with essential amino acids, B vitamins, and fiber, DDGS offers nutritional and economic advantages over traditional protein supplements. Its utilization could potentially address both the nutritional deficiencies in Haidong chicken rearing and the broader industry challenges of feed cost escalation.

Extensive research has documented the utilization of DDGS as a sustainable feed ingredient across livestock production systems. In ruminant nutrition, DDGS demonstrates dual benefits as both a rumen undegradable protein source and a modulator of microbial fermentation. By replacing soluble carbohydrates with their inherent fat and effective fiber, DDGS supplementation maintains rumen pH stability and reduces acidosis incidence compared to conventional diets (Reddy et al., 2021). Feeding trials in mutton sheep revealed that 10% corn DDGS inclusion improved average daily gain (ADG), enhanced carcass traits, and increased ruminal acetate production (Felix et al., 2012). However, excessive inclusion beyond 20% negatively impacts growth performance, underscoring the necessity for optimal dosage calibration (Cui et al., 2024). Similar benefits were observed in cows, where 15% barley DDGS enhanced serum biochemical indices and feed conversion ratio (Wilson et al., 2015). In swine production, strategic DDGS utilization yields species-specific outcomes of piglets; moderate inclusion promotes intestinal development, evidenced by greater villus height and increased Lactobacillus abundance (Stein and Shurson, 2009). Growing-finishing pigs fed moderate corn DDGS exhibited comparable growth performance to control groups while demonstrating a reduction in *Lawsonia intracellularis*-induced intestinal lesions (Li et al., 2022a). Notably, high inclusion levels alter fat composition, increasing polyunsaturated fatty acids (PUFA) in backfat, consequently reducing fat firmness (Swiatkiewicz et al., 2016). A critical analysis suggests a safe inclusion threshold of ≤24% corn DDGS for maintaining growth and carcass parameters in commercial swine operations (Rodriguez et al., 2021). DDGS is an excellent source of essential fatty acids, including linoleic acid, for poultry and can be used in combination with other feeds for breeding hens and laying hens. The addition of an appropriate amount of DDGS to feed can improve egg production performance, egg quality, serum biochemical parameters, and nutrient utilization rate in laying ducks (Ding et al., 2022).

However, currently, there are relatively few studies on the application of corn DDGS in the feeding of Haidong chickens. Therefore, this study aims to deeply analyze the effects of adding DDGS to the diet on the egg quality, serum physiological and biochemical, and immune indices of Haidong chickens. Furthermore, through microbial diversity sequencing, it analyzes the mechanisms of quality differences in Haidong chicken farming caused by DDGS to provide a theoretical and scientific basis for the rational utilization of DDGS in Haidong chicken farming, reducing farming costs, improving the production performance and product quality of Haidong chickens, and promoting the sustainable development of the local poultry industry. This multidisciplinary approach bridges critical knowledge gaps in unconventional feed resource applications, offering both theoretical insights and practical solutions for precision animal nutrition.

## Materials and methods

### Experimental design and sample collection

This experiment investigated local breed Haidong chickens aged 12 months (mid-laying period) from Qinghai Province; 288 healthy Haidong chickens with similar body weights were selected and divided into six groups: the control group (Con) and the experimental groups (E1–E5). There were 48 replicates in each group, and 12 laying hens were randomly selected for each replicate. The experiment was conducted under controlled environmental conditions at Huzhu Xueling Haidong Chicken Conservation Farm, employing standardized cage-rearing protocols with ad libitum access to feed and water. Based on the Chinese Agricultural Industry Standard of Chicken Feeding Standard (NY/T 33-2004), the nutrient requirements for metabolizable energy, crude protein, and calcium during the laying period are 2,800 kcal/kg, 16, and 3.8%, respectively. However, considering the smaller body size of Haidong chickens, we made appropriate adjustments to their dietary formulation. Detailed nutritional compositions of experimental formulations are presented in Table 1. The control group received a basal diet, while the experimental groups received isocaloric diets incorporating 2.5, 5, 7.5, 10, and 12.5% corn-derived DDGS as partial replacement for conventional corn-soybean meal substrates. The pre-feeding period of the experiment was 7 days, and the experimental period was 90 days. At the end of the 90-day experimental period, egg quality was analyzed. Feces were collected by replicate groups and were promptly preserved in collection tubes and placed in the sample tube collection box (with dry ice) to transport to the laboratory for microbial 16S rRNA sequencing.

**Table 1:** Ingredients and nutritional composition of experimental diets (% dry matter).

Ingredients (%)	D0 (0%)	D1 (2.5%)	D2 (5.0%)	D3 (7.5%)	D4 (10.0%)	D5 (12.5)
Corn	74.8	72.9	71.0	68.9	66.8	64.8
Soybean meal	15.0	14.3	13.8	13.4	13.0	12.7
Rapeseed meal	3.3	3.3	3.3	3.3	3.3	3.3
DDGS	0	2.5	5.0	7.5	10.0	12.5
Mountain flour	1.7	1.7	1.7	1.7	1.7	1.7
Yeast culture	0.4	0.4	0.3	0.3	0.3	0.3
Bran	1.0	1.1	1.0	1.0	1.0	1.0
Calcium bicarbonate	1.7	1.7	1.7	1.7	1.7	1.7
Salt	0.5	0.5	0.5	0.5	0.5	0.5
Vitamin-mineral premix^a^	1.7	1.7	1.7	1.7	1.6	1.6
Nutrient analysis, %						
ME, kcal/kg of diet	2,851	2,826	2,813	2,809	2,773	2,733
CP	15.1	15.3	15.7	15.8	15.9	16.1
Met	0.244	0.249	0.254	0.26	0.266	0.273
Lys	0.656	0.645	0.637	0.634	0.63	0.63
Ca	1.04	1.04	1.05	1.06	1.11	1.08
Available P	0.61	0.62	0.63	0.64	0.65	0.65
Crude fiber	2.3	2.4	2.6	2.7	2.9	3

a Provided the following per kilogram of diet with vitamin A 5,000 IU; vitamin D 1,000 IU; vitamin E 50 mg; vitamin K 1.40 mg; vitamin B1 1.80 mg; vitamin B2 8 mg; vitamin B6 4.10 mg; vitamin B12 0.01 mg; niacin 32 mg; calcium pantothenate 11 mg; folic acid 1.08 mg; biotin 0.18 mg; Fe 72 mg; Cu 7 mg; Zn 60 mg; Mn 90 mg.

### Hen productivity and egg quality

At the end of the trial, egg weight, albumen height, eggshell strength, egg yolk color, eggshell thickness, egg length, egg width, and Haugh unit (HU) were measured. Albumen heights were measured using a digital micrometer coupled to a tripod base. Diameters were measured using digital calipers. Eggshell thickness was measured in three regions (top, middle, and bottom) using a dial pipe gauge (FHK NFN380, Fujihira Industry Co., Ltd., Tokyo, Japan). Egg yolk color was ascertained using the Roche color fan (Hoffman-La Roche Ltd., Basel, Switzerland, where 15 = dark orange and 1 = light pale). Eggshell strength was estimated using an eggshell strength tester (FHK, Fujihira Industry Co., Ltd., Tokyo, Japan). HU values were calculated from egg weight (W) and albumen height (H), using the following equation as proposed by Drabik et al. (2021): HU = 100 log (H–1.7 W^0.37^ + 7.57).

### Serum biochemical parameters

Blood samples were collected from the brachial vein of chicken wings using sterile 21-gauge needles (10 mm × 25 mm) and transferred into 10 mL vacuum collection tubes. Following collection, samples were centrifuged at 3,000 rpm for 10 min at 4 °C to separate serum components. The resulting serum was aliquoted into sterile 0.5 mL polypropylene microcentrifuge tubes using calibrated pipettes and immediately frozen at −20 °C in temperature-monitored storage units until analysis. Serum biochemical parameters were quantified using commercial assay kits (Shanghai Youxuan Biotechnology Co., Ltd., China) following the manufacturer’s specifications. The specific operation steps are as described in the kit instructions, and the procedures were strictly followed. The content of alkaline phosphatase (ALP) (U/L), aspartate aminotransferase (AST) (U/L), alanine transaminase (ALT) (U/L), blood urea nitrogen (BUN) (mmol/L), creatinine (Cr) (mmol/L), progesterone (PROG) (ng/mL), growth hormone (GH) (ng/mL), estradiol (E2) (pg/mL), albumin (ALB) (g/L), globulin (GLB) (g/L), total protein (TP) (g/dL), glucose (GLU) (mmol/L), triglyceride (TG) (mmol/L), total cholesterol (TC) (mmol/L), low density lipoprotein (LDL) (mmol/L), low density lipoprotein cholesterol (LDL-C) (mmol/L), high density lipoprotein (HDL) (mmol/L), high density lipoprotein cholesterol (HDL-C) (mmol/L), interferon-gamma (IFN-γ) (pg/mL), interleukin-6 (IL-6) (pg/mL), interleukin-10 (IL-10) (pg/mL), tumor necrosis factor-α (TNF-α) (pg/mL), immunoglobulin A (IgA) (μg/mL), immunoglobulin G (IgG) (mg/mL), immunoglobulin M (IgM) (μg/mL) were determined using commercial ELISA kit.

### Microbial 16S rRNA sequencing

Microbial DNA was extracted using the HiPure Soil DNA Kits (or HiPure StoolDNA Kits) (Magen, Guangzhou, China) according to the manufacturer’s protocols. The 16S rDNA V3–V4 region of the ribosomal RNA gene was amplified by PCR (94 °C for 2 min, followed by 30 cycles at 98 °C for 10 s, 62 °C for 30 s, and 68 °C for 30 s and a final extension at 68 °C for 5 min) using primers 341F: CCTACGGGNGGCWGCAG; 806R: GGACTACHVGGGTATCTAAT. PCR reactions were performed in a 50 μL mixture containing 5 μL of 10 × KOD Buffer, 5 μL of 2 mM dNTPs, 3 μL of 25 mM MgSO_4_, 1.5 μL of each primer (10 μM), 1 μL of KOD Polymerase, and 100 ng of template DNA. Amplicons were extracted from 2% agarose gels and purified using the AxyPrep DNA Gel Extraction Kit (Axygen Biosciences, Union City, CA, United States) according to the manufacturer’s instructions and quantified using the ABI StepOnePlus Real-Time PCR System (Life Technologies, Foster City, United States). Purified amplicons were pooled in equimolar and paired-end sequenced (2 × 250) on an Illumina platform according to the standard protocols. Raw data containing adapters or low-quality reads would affect the following assembly and analysis. Thus, to obtain high-quality, clean reads, raw reads were further filtered according to the following rules using FASTP (Chen et al., 2018). Clean tags were searched against the reference database (version r2011051) to perform reference-based chimera checking using the UCHIME algorithm (Edgar et al., 2011). The effective tags were clustered into operational taxonomic units (OTUs) at ≥97% similarity using the UPARS (version 9.2.64) pipeline. The representative sequences were classified into organisms by a naive Bayesian model using the RDP classifier (version 2.2) based on the SILVA database (version 132), the Greengene database (version gg_13_5), the UNITE database (version 8.0), or the ITS2 database (version update_2015), with the confidence threshold values ranging from 0.8 to 1. The abundance statistics of each taxonomy were visualized using Krona (version 2.6) (Ondov et al., 2011). Chao1, Simpson, and all other alpha diversity indices were calculated in QIIME (version 1.9.1). Alpha index comparisons between groups were calculated by Welch’s *t*-test and the Wilcoxon rank test in the R project Vegan package (version 2.5.3). The KEGG pathway analysis of the OTUs was inferred using Tax4Fun (version 1.0) or PICRUSt (version 2.1.4). Microbiome phenotypes of bacteria were classified using BugBase. The FAPROTAX database (Functional Annotation of Prokaryotic Taxa) and associated software (version 1.0) were used to generate ecological functional profiles of bacteria. The functional group of the fungi was inferred using FUNGuild (version 1.0). Analysis of function difference between groups was calculated by Welch’s *t*-test, the Wilcoxon rank test, the Kruskal–Wallis *H* test, and Tukey’s HSD test in the R project Vegan package (Asshauer et al., 2015) (version 2.5.3).

### Data analysis

Statistical analyses were performed using SPSS Statistics 22 (SPSS Inc., Chicago, IL, United States). One-way analysis of variance (ANOVA) was conducted to evaluate the effects of graded dietary inclusion levels of DDGS on egg quality parameters and serum biochemical profiles in Haidong chickens. Quantitative data are presented as mean ± standard error of the mean (SEM). Comparisons of group means were performed using Duncan’s multiple range test at a significance threshold of *p* < 0.05. All experimental treatments were independently replicated three times.

## Results

### Hen performance

There was no difference in egg mass of laying hens between the groups. Egg weight significantly increased with the dietary inclusion level of DDGS (*p* < 0.05); however, there were no differences observed among the D2, D3, D4, and D5 groups. The feed intake of D3, D4, and D5 groups was significantly lower than that of the control group (*p* < 0.05). The D4 group exhibited the lowest FCR, indicating improved feed efficiency. Egg production in D3, D4, and D5 groups was significantly higher compared to the control group (*p* < 0.05) (Table 2).

**Table 2:** The overall performance of Haidong hens fed diets containing DDGS.

Items	Treatments						SEM	*p*-value (CON vs. DDGS)
	D0 (0% DDGS)	D1 (2.5% DDGS)	D2 (5.0% DDGS)	D3 (7.5% DDGS)	D4 (10.0% DDGS)	D5 (12.5% DDGS)		
Egg mass	40.75^a^	41.00^a^	41.50^a^	41.70^a^	40.15^a^	41.24^a^	1.841	0.25
Egg weight, g	47.98^c^	50.60^b^	52.88^a^	52.20^a^	52.50^a^	52.48^a^	1.467	0.01
Feed intake, g/h/d	74.40^a^	72.30^c^	74.98^a^	73.10^b^	73.30^b^	73.23^b^	1.375	0.001
FCR	1.50^ab^	1.60^a^	1.52^ab^	1.50^ab^	1.37^b^	1.54^ab^	0.397	<0.001
Egg production, %	34.1^b^	35.74^a^	34.5^b^	35.1^a^	35.4^a^	35.8^a^	1.056	0.02

^a–c^Means with different superscripts within each variable are significantly different at *p* < 0.05; Tukey’s tests were applied to compare means. SEM, standard error of the mean; FCR, feed conversion ratio.

### Dynamic changes in egg quality of Haidong chickens under graded dietary inclusion of DDGS

**Figure 1:**
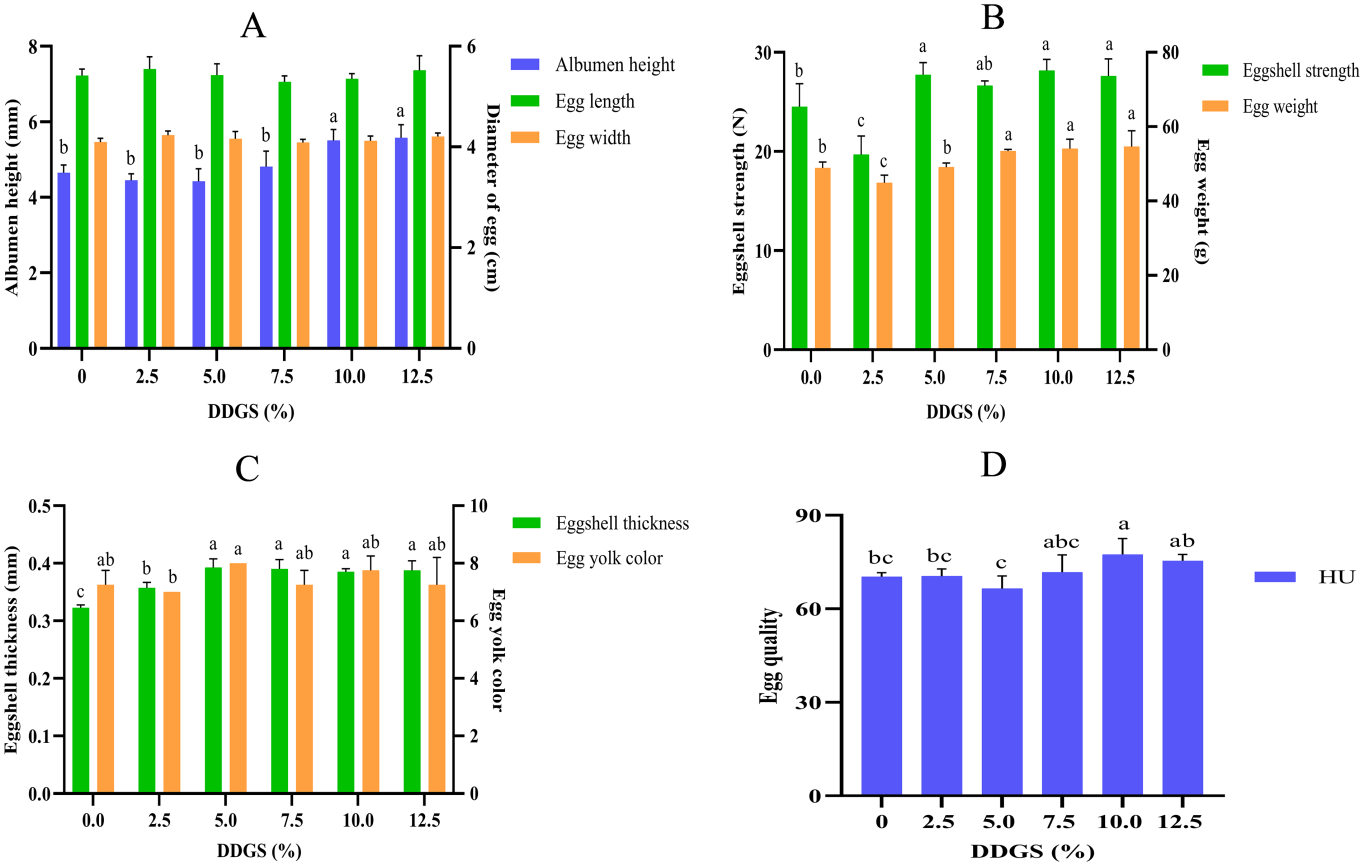
Effect of dietary DDGS on egg quality of Haidong chickens. HU, Haugh unit. ^a–c^Values within groups at graded dietary inclusion of DDGS with no common superscripts differ significantly (*p* < 0.05). The effects of DDGS supplementation on the serum biochemical profiles of Haidong chickens.

**Figure 2:**
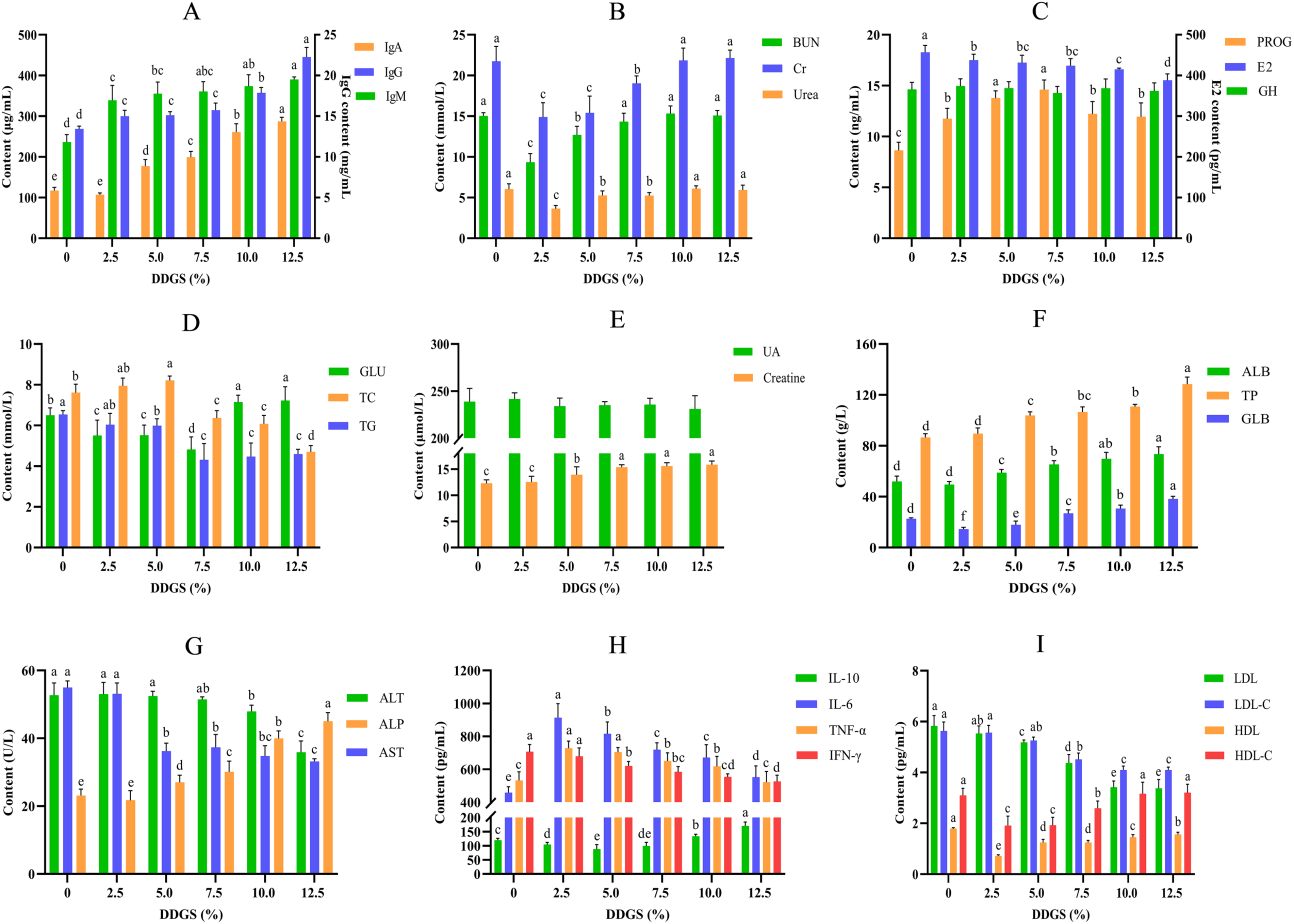
The effects of DDGS supplementation on the serum biochemical profiles of Haidong chickens. ^a–d^Values within groups at graded dietary inclusion of DDGS with no common superscripts differ significantly (*p* < 0.05).

The egg quality of Haidong chicken was revealed to have dose-dependent responses to DDGS supplementation (Figure 1). Quantitative analysis indicated dimensional stability in egg quality, with egg diameter maintaining consistency (*p* > 0.05) across all dietary DDGS inclusion levels (0–12.5% DDGS) in Haidong chickens. Albumen height increased by 14.4% at 7.5–10.0% DDGS inclusion levels (*p* < 0.05). This increment was bookended by stabilization phases showing nonsignificant variation at both lower (0–7.5% DDGS) and higher (10.0–12.5% DDGS) supplementation ranges, as depicted in Figure 2A. Compared with the CON group, eggshell strength was significantly decreased in the group that consumed a diet with 2.5% DDGS (*p* < 0.05), subsequently increased from 2.5% DDGS to 5.0% DDGS (*p* < 0.05), but became insignificant from 5.0% DDGS to 12.5% DDGS. The egg weight decreased first, then increased with increasing DDGS addition. It significantly increased from 2.5% DDGS to 7.5% DDGS (*p* < 0.05), but became insignificant from 7.5% DDGS to 12.5% DDGS (Figure 1B). The eggshell thickness significantly increased from 0–5.0% DDGS inclusion levels (*p* < 0.05). No significant variance was observed in eggshell thickness between the group that consumed a diet with 5.0% DDGS to 12.5% DDGS. The egg yolk color was higher in the 5.0% DDGS diet group compared with other groups, except for the group with a 2.5% DDGS diet group. There were no significant differences in yolk color compared with the other groups (Figure 1C). The eggs in the 10% DDGS group had the highest Haugh unit (HU) value (Figure 1D).

### Blood biochemical constituents

**Figure 3:**
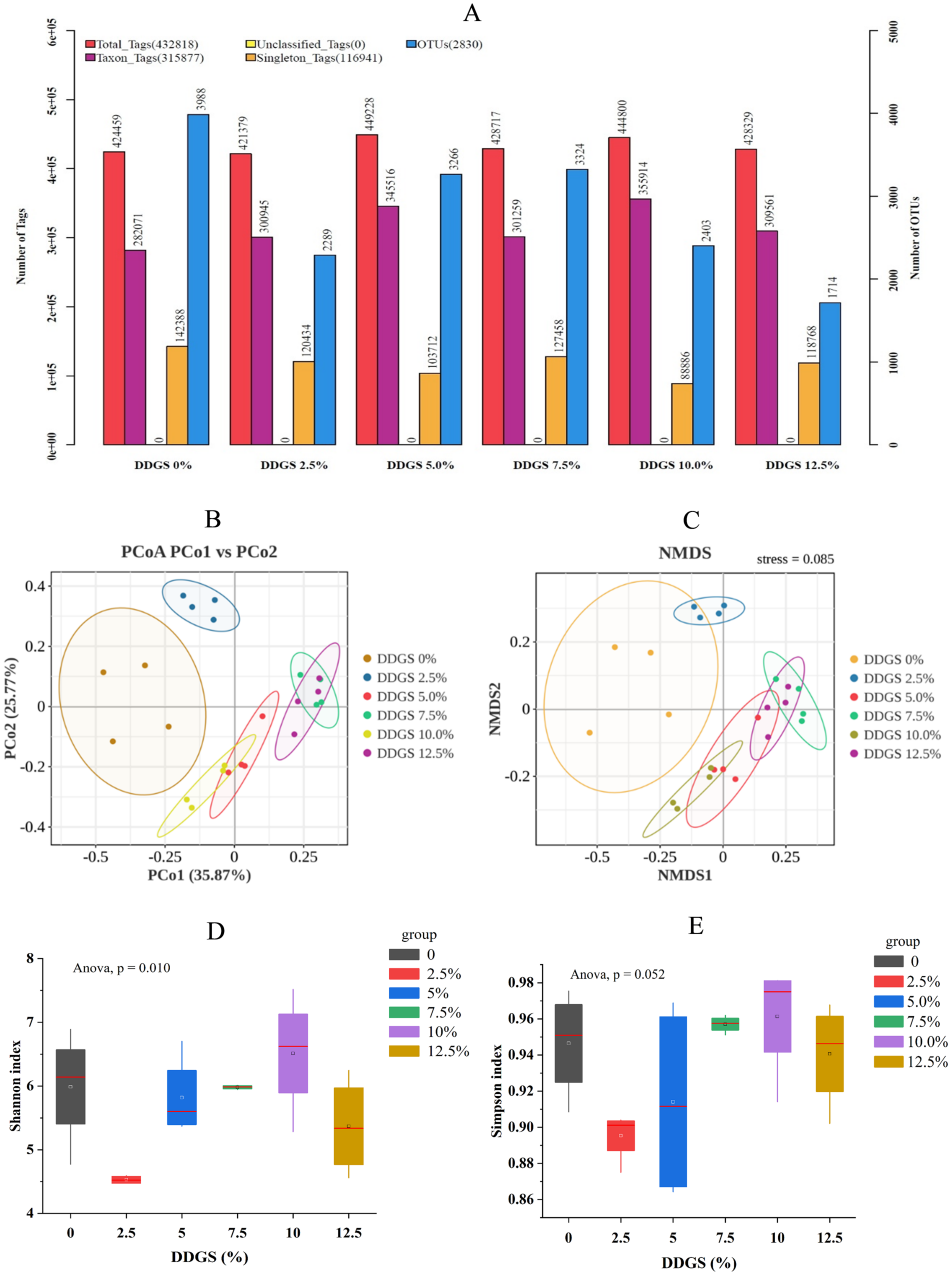
Comparative analysis of gut microbial species diversity in Haidong chicken with different DDGS supplementation. **(A)** Statistical chart of Tags and OTUs counts. **(B)** Principal coordinate analysis. **(C)** Non-metric multidimensional scaling. **(D,E)** Microbial diversity indicators (Shannon and Simpson indices) in Haidong chicken with different DDGS supplementation.

A comprehensive serum biochemical profiling was conducted on Haidong chicken using standardized spectrophotometric assays (Figure 2). The levels of IgM, IgA, and IgG increased with the increase of dietary inclusion of DDGS. Moreover, serum IgG and IgA exhibited significantly higher concentrations in the 12.5% DDGS supplementation group compared to other treatment groups (*p* < 0.05; Figure 2A). Serum biomarkers of nitrogen metabolism (BUN, Cr and Urea) exhibiting initially increased and then decreased with increasing dietary inclusion of DDGS, initial depression was observed at 2.5% inclusion DDGS, however, they subsequently increased with further addition of DDGS, reaching their highest at 12.5% DDGS inclusion (Figure 2A). There was no significant variance in GH between the control group and the groups that received DDGS treatments (*p* > 0.05). The level of E2 decreased with the increasing content of DDGS in the diet, and the lowest E2 level was observed in the group that consumed a diet with 12.5% DDGS (*p* < 0.05). Meanwhile, the level of PROG initially increased and then decreased with the increasing content of DDGS in the diet, reaching its highest level in the group that consumed a diet with 7.5% DDGS (Figure 3C). Serum GLU levels in Haidong chickens demonstrated a U-shaped trajectory with increasing dietary DDGS inclusion, reaching the lowest concentration at 7.5% DDGS supplementation. Conversely, total TC exhibited an inverted U-shaped response; it was lowest in the group that consumed a diet with 12.5% DDGS (*p* < 0.05). Serum TG showed a progressive decrease across the DDGS supplementation gradient, indicative of enhanced lipid catabolism (Figure 3D). Furthermore, the effect of dietary DDGS on serum UA and creatine in Haidong chickens was investigated (Figure 2E). Dietary DDGS supplementation exhibited no significant effect on serum UA levels in Haidong chickens (*p* > 0.05). Conversely, serum creatine concentrations increased progressively with incremental DDGS inclusion, reaching the highest levels at the 12.5% DDGS supplementation level (*p* < 0.05). Additionally, the levels of TP and ALB increased with the incremental inclusion of DDGS in the diet. Conversely, the content of GLB initially decreased before increasing with the incremental inclusion of DDGS in the diet. Notably, the highest of TP and GLB was observed in the group that consumed a diet with 12.5% DDGS (*p* < 0.05; Figure 2F). Furthermore, the level of ALT and AST decreased with the increasing content of DDGS in the diet. However, the level of ALP increased with the increasing content of DDGS in the diet and reached its highest level in the group that consumed a diet with 12.5% DDGS (*p* < 0.05; Figure 2G). The changes in serum cytokines in Haidong chicken DDGS supplementation in the diet were analyzed (Figure 2H). Serum cytokines changed with the incremental inclusion of DDGS in the diet. Serum IL-6, TNF-α, and IFN-γ initially increased and then decreased with increasing DDGS addition; conversely, the anti-inflammatory cytokine IL-10 initially decreased and then increased as the DDGS addition increased. Furthermore, lipoproteins were also analyzed in the study, as depicted in Figure 2I. The levels of LDL and LDL-C decreased with the increasing content of DDGS in the diet. Conversely, the levels of HDL and HDL-C initially decreased and then increased with the increasing content of DDGS, reaching their highest levels at 12.5% DDGS supplementation.

### Changes in gut microbiota succession with different DDGS supplementation

Illumina sequencing of the 16S rRNA gene V3–V4 regions yielded an average of 432,818 raw reads per experimental group. Following stringent quality control, the retained high-quality sequences revealed distinct OTU profiles across DDGS supplementation gradients: 3,988 (0% DDGS), 2,289 (2.5% DDGS), 3,266,192 (5.0% DDGS), 3,324 (7.5% DDGS), 2,403,167 (10.0% DDGS), and 1,714 OTUs (12.5% DDGS), respectively (Figure 3A). Principal coordinate analysis (PCoA) and non-metric multidimensional scaling (NMDS) were used to cluster the samples according to the composition of microbial communities to visually display the similarities and differences between samples. PCoA and NMDS showed changes in gut microbes of Haidong chicken with different DDGS supplementation to the diet (Figures 3B,C). Alpha-diversity assessment revealed a dose-dependent modulation of gut microbial complexity. The Shannon index exhibited a biphasic response, with the 2.5% DDGS group showing significantly reduced diversity compared to controls, reaching its highest at 10% DDGS supplementation (*p* < 0.05; Figure 3D). The Simpson index exhibited a similar trend to the Shannon index across different groups, but no significant differences were observed (Figure 3E).

**Figure 4:**
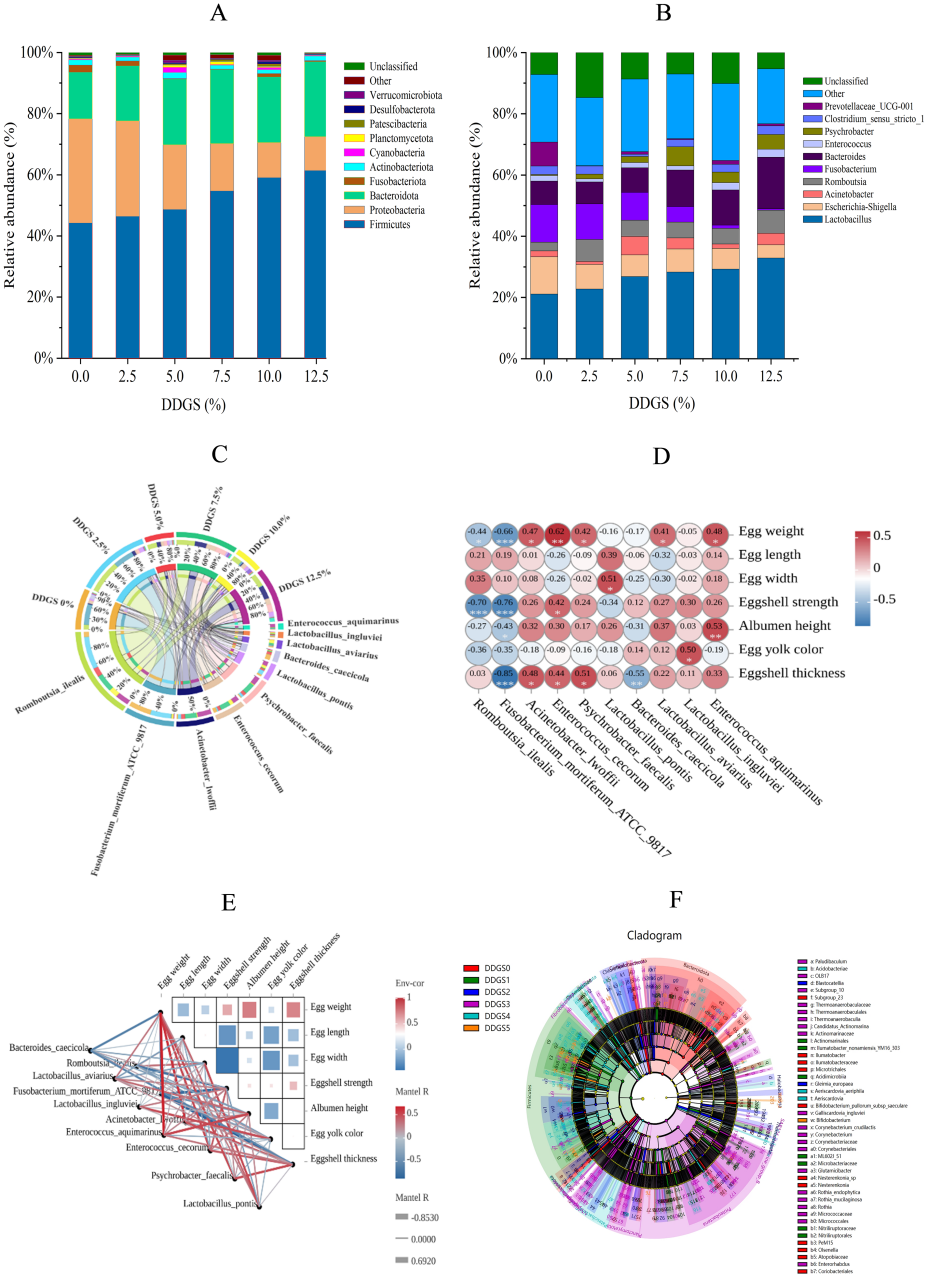
Rumen microbial composition analysis of Haidong chickens with different DDGS supplementation. **(A)** The relative abundance of bacteria at the phylum level. **(B)** The relative abundance of bacteria at the genus level. **(C)** Circos plot of species distribution. **(D,E)** Correlation analysis between gut microorganisms and egg quality. ^*^Correlation was significant at *p* < 0.05. ^**^Correlation was significant at *p* < 0.01. Env-cor, environments-correlation. Red represents positive correlation, and blue represents negative correlation. The color intensity is proportional to the correlation value. **(F)** LEfSe analysis. DDGS0 = 0% DDGS supplementation; DDGS1 = 2.5% DDGS supplementation; DDGS2 = 5.0% DDGS supplementation; DDGS3 = 7.5% DDGS supplementation; DDGS4 = 10.0% DDGS supplementation; DDGS5 = 12.5% DDGS supplementation.

Microbiota composition profiling identified Firmicutes, Bacteroidota, and Proteobacteria as the predominant bacterial phyla in the gut microbiome of Haidong chickens following dietary supplementation with DDGS (Figure 4A). Firmicutes abundance demonstrated a progressive increase from 44.16% in Con to 61.32% in the 12.5% DDGS group (*p* < 0.05), with the highest DDGS supplementation group showing significant divergence from other groups. Conversely, Proteobacteria displayed an inverse correlation with the increase of DDGS supplementation (*p* < 0.05), declining from 34.08% (Con) to 11.11% (12.5% DDGS). Nevertheless, the dominant abundance of Bacteroidota increased significantly with increasing DDGS supplementation (*p* < 0.05) compared with the control group (15.26%). The abundance of Bacteroidota was significantly higher in the group than in the 12.5% DDGS group (24.51%) (*p* < 0.05). Genus-level compositional dynamics demonstrated pronounced DDGS dose responsiveness (Figure 4B). *Lactobacillus* abundance exhibited a significant positive correlation with DDGS supplementation levels (*p* < 0.05), escalating from 21.12% in Con to 32.86% at the 12.5% DDGS group (*p* < 0.05). Conversely, *Escherichia-Shigella* abundance decreased with the increase of DDGS supplementation, and the lowest abundance of *Escherichia-Shigella* was observed in the group that consumed a diet with 12.5% DDGS (4.28%). Notably, *Romboutsia* abundance was lower in the control group (2.83%) than in all other groups. *Fusobacterium* abundance showed a significant decrease with the increase of DDGS supplementation (*p* < 0.05), and *Bacteroides* abundance showed a significant increase with the increase of DDGS supplementation (*p* < 0.05). According to the Circos analysis, dietary supplementation of DDGS in Haidong chickens promoted the growth of *Lactobacillus pontis* and *Romboutsia ilealis*. These microbes could inhibit harmful gut microbes and pathogens, produce short-chain fatty acids (SCFAs), and play a critical role in maintaining intestinal barrier function, regulating host energy metabolism, and modulating immune responses (Figure 4C). To understand the effects of gut microbiota on egg quality parameters, Pearson’s analysis was carried out between 7 egg-quality parameters and 10 microorganisms related to the amino acid metabolism of Haidong chickens fed with different levels of DDGS. The results showed that *Romboutsia ilealis* had a significantly negative correlation with eggshell strength and *Enterococcus cecorum* had a significantly positive correlation with egg weight (Figures 4D,E). LEfSe analysis identified distinct biomarkers associated with varying levels of DDGS supplementation in the Haidong chicken diet (Figure 4F).

**Figure 5:**
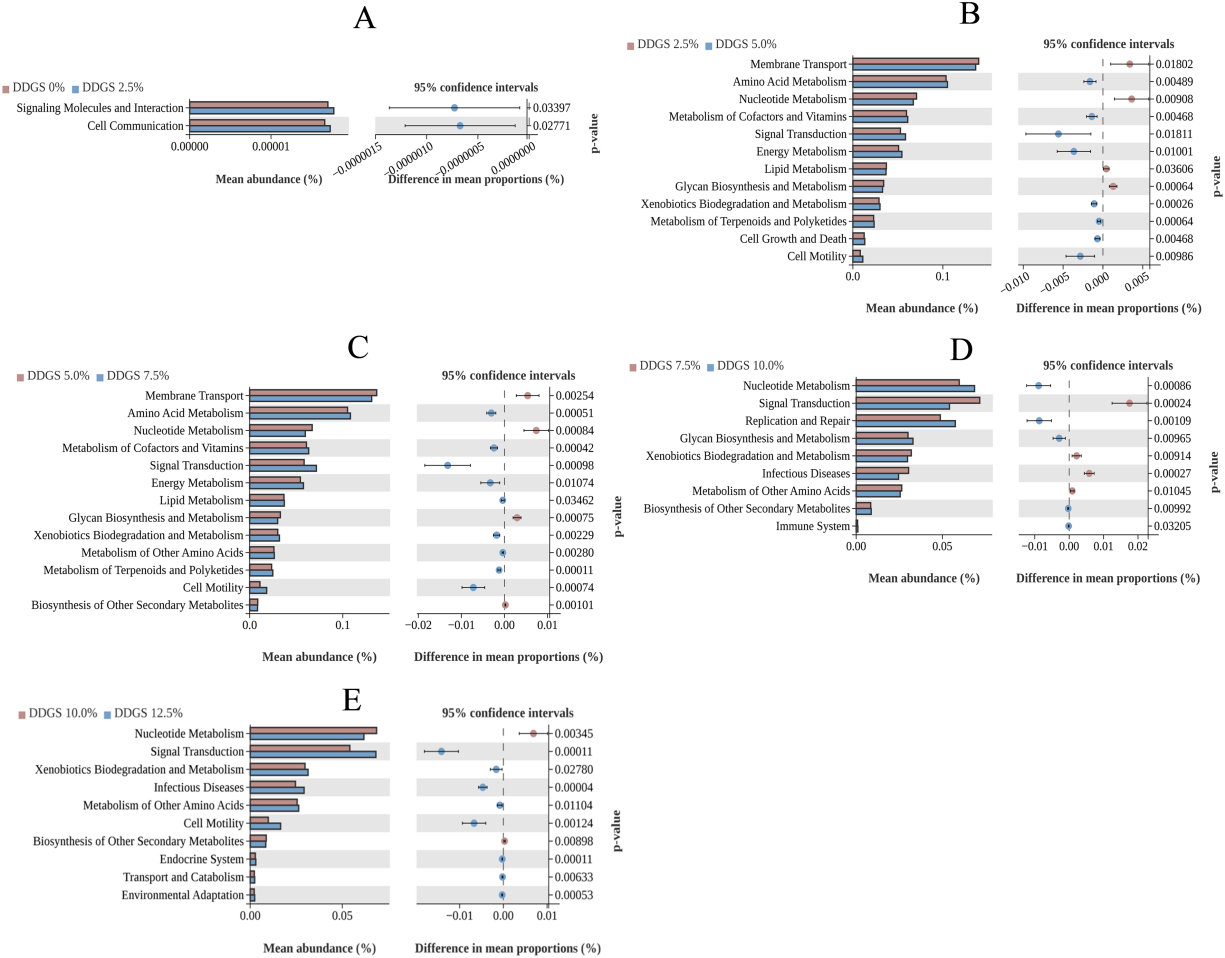
Gut microbial function through PICRUSt2 analysis of Haidong chickens with different DDGS supplementation.

Moreover, differential microbial KEGG functional analysis revealed the following trends in Haidong chickens with varying levels of DDGS supplementation in the diet. KEGG functional analysis revealed that 2.5% DDGS induced significant up-regulation of cell communication pathways and signaling molecule interactions in Haidong chickens (Figure 5A). 12.5% DDGS triggered enhanced amino acid metabolism, energy metabolism, and metabolism of cofactors and vitamins (Figure 5B). 7.5% DDGS further amplified amino acid metabolism, energy metabolism, and the metabolism of other amino acids in Haidong chickens (Figure 5C). 10.0% DDGS demonstrated immunomodulatory effects via suppression of infectious disease pathways coupled with innate immune potentiation (Figure 5D). 12.5% DDGS optimized environmental adaptation through flagellar assembly and two-component signal transduction systems while up-regulating stress-responsive amino acid metabolism (Figure 5E).

## Discussion

Dietary protein plays a critical role in the development of the digestive system and in enhancing growth performance. DDGS, a co-product of ethanol production derived primarily from the fermentation of corn, serves as a valuable protein-rich feed ingredient. Corn DDGS was characterized by its high content of digestible protein, metabolizable energy, essential minerals, fat-soluble vitamins, and bioactive compounds that synergistically support nutrient absorption and growth efficiency in livestock (Vardhan Reddy et al., 2024). Consequently, DDGS has been extensively incorporated into livestock diets as a strategic feed ingredient, with well-documented applications in swine, ruminant, and poultry production systems. Peer-reviewed studies demonstrate its successful utilization at inclusion rates up to 30% in corn-soybean meal-based diets, capitalizing on its balanced amino acid profile and fermentable fiber content that align with the digestive physiology of monogastric and polygastric species (Reddy et al., 2021; Li et al., 2022a). A growing body of evidence indicates that the strategic incorporation of DDGS in layer rations significantly optimizes feed cost-efficiency while enhancing egg production performance. Lee et al. (2008) demonstrated that dietary incorporation of DDGS at graded inclusion levels (0, 10, 15, and 20%) in 24-week-old Hy-Line laying hens maintained egg production parameters within industry benchmarks, while reducing feed costs by 12–15% through partial replacement of soybean meal. Roberson et al. (2005) demonstrated that dietary inclusion of DDGS at graded levels (0, 5, 10, and 15%) elicited dose-dependent reductions in egg production during specific phases of the late laying period (48–56 weeks of age). Notably, hens fed DDGS exhibited significant declines in laying performance at 52–53 weeks of age, with transient production losses also observed at 51 and 53 weeks. The species- and age-specific effects of DDGS incorporation in poultry diets are evident across studies. While Ruan et al. (2018) reported no adverse impacts on laying performance in Longyan ducks (17-week-old) fed diets containing up to 30% DDGS, our findings in Haidong hens align more closely with observations in layer chickens. While Lumpkins et al. (2005) and El-Gabry et al. (2010) independently showed 6–9% improvements in eggshell metrics at 15% inclusion levels in White Leghorn and Inshas hens, respectively. In the current study, dietary DDGS supplementation elicited distinct temporal patterns in Haidong egg quality. The absence of effects on egg diameter contrasts with the progressive increases observed in albumen height and eggshell thickness, consistent with previous reports of DDGS-induced calcium retention enhancement. However, the biphasic response in eggshell strength at 2.5% DDGS supplementation and egg weight fluctuations suggests a critical threshold effect. This transitional phase at low inclusion levels may reflect incomplete intestinal microbiota colonization, as evidenced by reduced *Lactobacillus* spp. Abundance, potentially impairing mineral absorption efficiency during early adaptation. The parabolic trends in yolk color and HU mirror the dual-phase carotenoid metabolism initial enhancement through DDGS-derived xanthophyll, followed by antioxidant-prooxidant imbalance at higher inclusions. These nonlinear responses underscore the necessity for precision formulation strategies.

Serum biochemical parameters serve as valuable biomarkers for evaluating nutritional status and systemic health. Comparative analysis revealed significant differences in serum immunoglobulin (IgA, IgG, and IgM) levels between the control and treatment groups. Notably, a positive correlation was observed between dietary DDGS concentration and serum immunoglobulin levels, with IgA, IgG, and IgM concentrations showing dose-dependent increases. The experimental data clearly demonstrate that dietary supplementation with DDGS significantly enhances total serum immunoglobulin (IgA, IgM, and IgG) concentrations in Haidong chickens, confirming and extending previous observations in this field (Barekatain et al., 2013). BUN is a crucial biomarker of protein catabolism and renal filtration efficiency and serves as a key diagnostic marker for assessing glomerular function. In the current investigation, serum BUN concentrations showed no statistically significant differences between experimental groups receiving 10 and 12.5% DDGS supplementation and the Con. These findings align with previous work by Hong et al. (2001), who demonstrated comparable null effects on porcine serum BUN levels following dietary supplementation with Yucca extract and/or far-infrared emitting materials in finishing swine. The consistent absence of BUN alterations across these nutritional interventions suggests preserved renal filtration capacity and stable nitrogen metabolism under these dietary modification conditions. Cr is a nitrogenous compound formed through the catabolism of phosphocreatine in skeletal muscle and serves as a reliable biomarker for assessing glomerular filtration rate (GFR) and renal functional integrity. Serum Cr concentrations, regulated primarily by renal excretion, reflect kidney filtration efficiency and are clinically utilized to evaluate renal health. In this study, no significant differences in serum BUN levels were observed among groups supplemented with 7.5, 10%, or 12.5% DDGS compared to the control group. Notably, serum urea concentrations in the DDGS diet Haidong chickens displayed a biphasic pattern of initial reduction followed by a gradual elevation, yet remained consistently lower than control levels across all supplementation phases. These findings align with prior research by Wang et al. (2021) and Cui et al. (2023), who reported analogous stability in nitrogen metabolite profiles under modified dietary regimens, suggesting preserved renal homeostasis despite nutritional interventions. This study revealed a biphasic response in serum PROG levels of Haidong chickens, characterized by an initial elevation followed by progressive decline with increasing dietary DDGS inclusion levels. This dynamic pattern suggests DDGS supplementation may modulate endocrine pathways to enhance laying performance, potentially through phytoestrogenic interactions with reproductive hormone regulation. The observed PROG fluctuations align with findings from swine nutrition research, which shows that dietary supplementation with 200 mg/kg daidzein significantly elevated serum progesterone concentrations in gestating sows at day 35 of pregnancy (Li et al., 2021). This phytoestrogenic activity may directly influence ovarian steroidogenesis and hypothalamic–pituitary-gonadal axis signaling, ultimately affecting serum progesterone dynamics in laying hens. Serum E2 concentrations exhibited a progressive decline in correlation with escalating dietary DDGS inclusion levels, a phenomenon that closely parallels the dose-dependent estrogenic modulation patterns reported by Akiba et al. (1982) in their investigation of phytoestrogen-rich feed additives. The present study demonstrated that dietary supplementation with DDGS showed no significant effect on serum GH concentrations in Haidong chickens. This finding contrasts with observations by Sibel et al. (2018) in ovine models, where DDGS inclusion significantly elevated circulating GH levels in ewes. This species-specific divergence may be attributed to physiological differences in metabolic regulation during the reproductive phase, notably, the inherently slow growth rate characteristic of Haidong chickens during peak lay periods, which likely reduces growth-related endocrine demands. Serum glucose serves as a primary energy substrate for physiological functions. Our findings demonstrate that dietary supplementation with 12.5% DDGS induced a significant elevation in circulating glucose levels, reaching peak concentrations compared to other experimental formulations. Li et al. (2022b) conducted a systematic investigation of dietary DDGS supplementation in Lande geese, demonstrating a non-significant positive correlation between DDGS inclusion levels and serum GLU concentrations across treatment groups. TG is a fundamental constituent of lipid metabolism, which exhibited an inverse relationship with DDGS inclusion levels in this study. This observed dose-dependent reduction in serum TG concentrations aligns with the hematological patterns reported in Lande geese fed DDGS-supplemented diets (Li et al., 2022b). These collective findings suggest DDGS might possess lipid-modulating properties, potentially serving as a functional feed ingredient for TG regulation. Total TC, a clinical biomarker representing the sum of free cholesterol and esterified cholesterol fractions in circulation, exhibited a biphasic response to DDGS supplementation in Haidong chickens. Serum TC concentrations initially increased at moderate DDGS inclusion levels before declining at higher dietary proportions. Notably, DDGS supplementation demonstrated no statistically significant effect on serum UA concentrations, suggesting distinct metabolic pathways regulate these biochemical parameters. Creatine functions as a critical mediator of cellular energy homeostasis by integrating into the phosphagen system. This nitrogenous organic acid reversibly phosphorylates adenosine triphosphate (ATP) via creatine kinase catalysis, forming creatine phosphate. During muscle contraction, the phosphagen system rapidly regenerates ATP through phosphate group transfer from creatine phosphate to adenosine diphosphate (ADP), ensuring immediate energy provision (Bonilla et al., 2021). Dietary inclusion of DDGS induced a marked elevation in serum creatine concentrations in Haidong chickens, suggesting this nutritional intervention might potentiate neuromuscular performance through enhanced phosphagen system functionality. These findings corroborate the mechanistic relationship between dietary amino acid availability and muscle creatine synthesis established in recent poultry nutrition research, particularly aligning with Aguihe’s et al. (2022) investigation into threonine-glycine equilibrium effects on broiler muscle creatine dynamics. Serum TP, comprising ALB and GLB fractions, serves critical homeostatic functions, including colloidal osmotic pressure maintenance, metabolite transport, and physiological regulation of blood pH. Our findings revealed a significant elevation in both ALB and TP concentrations following DDGS supplementation. This protein-mediated response likely stems from DDGS’s high crude protein content, which enhances hepatic biosynthesis of transport proteins. Notably, GLB concentrations exhibited a biphasic pattern, suggesting dynamic modulation of immunoglobulin synthesis during dietary adaptation. These observations align with Hu’s et al. (2021) report on energy-dependent protein metabolism in broilers. Serum ALT is a hepatocyte-localized enzyme integral to amino acid metabolism and serves as a sensitive biomarker of hepatocellular integrity. Compromised hepatic membranes facilitate ALT release into circulation, elevating serum levels proportionally to parenchymal damage. Similarly, AST functions as a mitochondrial transaminase in hepatocytes and cardiomyocytes, catalyzing key reactions in the malate–aspartate shuttle and gluconeogenesis pathways. Notably, dietary supplementation with DDGS significantly reduced serum ALT and AST concentrations in Haidong chickens compared to controls. This hepatoprotective effect, evidenced by attenuated transaminase leakage, aligns with hepatic function preservation observed in DDGS-fed geese (Li et al., 2022b), suggesting conserved mechanisms of nutrient-mediated liver homeostasis across poultry species. IL-6 and TNF-α constitute central orchestrators of innate immunity, functioning as pleiotropic inflammatory mediators that coordinate both acute-phase responses and chronic inflammatory cascades through JAK/STAT and NF-κB signaling pathways, respectively (Ciobanu et al., 2020). IL-10 functions as a pleiotropic anti-inflammatory cytokine that orchestrates immune homeostasis through transcriptional suppression of pro-inflammatory mediators. Mechanistically, IL-10 signaling via the STAT3 pathway inhibits nuclear factor-κB (NF-κB) activation in activated macrophages and synovial fibroblasts, thereby down-regulating synthesis of IL-6 and TNF-α at both transcriptional and post-translational levels (Wang et al., 2019; York et al., 2024). Dietary supplementation with DDGS induced a significant elevation in serum IL-10 levels in Haidong chickens, which subsequently mediated the downregulation of pro-inflammatory cytokines IL-6 and TNF-α through JAK/STAT3 signaling pathway modulation. This immunoregulatory cascade effectively attenuated systemic inflammatory responses, as evidenced by reduced hepatic acute-phase protein synthesis and improved clinical inflammation markers. Serum LDL plays a critical role in cholesterol mobilization by delivering endogenous cholesterol to peripheral tissues, while HDL facilitates reverse cholesterol transport via the hepatic scavenger receptor-B1 pathway, effectively mitigating atherogenic risks by preventing cholesterol deposition in vascular endothelia. In avian models, elevated serum LDL-C serves as a hallmark of dyslipidemia, correlating with impaired hepatic LDL receptor activity. Conversely, HDL-C is the sterol fraction bound to apolipoprotein A1 in HDL particles and exhibits vasculoprotective properties by promoting cholesterol efflux from macrophages and enhancing hepatic catabolism and biliary excretion. This lipoprotein-mediated cholesterol trafficking equilibrium is indicative of systemic lipid homeostasis, with elevated HDL-C:LDL-C ratios reflecting optimal metabolic health in poultry (Nieddu et al., 2022; Yelamanchili et al., 2025). It indicates that dietary supplementation with DDGS significantly enhanced hepatic metabolic homeostasis in Haidong chickens.

The compositional diversity of gut microbiota has been extensively documented as a critical determinant in host environmental adaptation. In avian species, the chicken intestinal ecosystem harbors a remarkably complex multi-kingdom consortium encompassing bacterial communities, archaeal populations, protozoan species, fungal elements, and bacteriophage populations. This intricate biological network constitutes one of nature’s most efficient digestive systems, demonstrating unparalleled metabolic versatility and symbiotic integration across taxonomic domains (Ma et al., 2023; Ma et al., 2024). The current study showed significant dose-dependent effects of DDGS on gut microbial ecology in Haidong chickens. Notably, the 2.5% DDGS group exhibited a marked reduction in Shannon diversity indices compared to controls, with subsequent analysis demonstrating a non-linear response pattern; microbial diversity initially increased before declining with the increase of DDGS addition, peaking at 10% DDGS supplementation. This optimal concentration corresponded with enhanced microbial community structure, as evidenced by increased relative abundance and α-diversity metrics, potentially attributable to DDGS-induced improvements in feed utilization efficiency and microbiome reorganization. The dose-dependent fluctuations in α-diversity align with the framework established by recent microbiome interaction models (Chandrasekaran et al., 2024). Interestingly, β-diversity analysis through Simpson indices remained stable across all treatment groups, suggesting preserved community evenness despite altered species richness. Notably, even at the maximum supplementation rate of 12.5%, DDGS administration maintained microbial community integrity without compromising taxonomic richness or ecological diversity. Phylogenetic profiling confirmed the expected predominance of Bacteroidetes and Firmicutes phyla, consistent with previous characterizations of avian gut ecosystems (Zhao et al., 2022; Shen et al., 2024), indicating maintenance of core microbial functions despite dietary interventions. The gut microbiota orchestrates host metabolic homeostasis through transcriptional regulation of nutrient-processing genes, employing multifaceted mechanisms ranging from enzymatic biotransformation to epigenetic modification of metabolic pathways (Rowland et al., 2018). The present study demonstrated that dietary incorporation of DDGS induced significant phylum-level restructuring of the gut microbiota, notably elevating the relative abundance of Firmicutes and Bacteroidetes compared to control cohorts. These microbial consortia functionally mediate polysaccharide metabolism through enhanced glycoside hydrolase activity and butyrate biosynthesis, thereby facilitating energy harvest efficiency and xenobiotic biotransformation via conjugation pathways (Pont et al., 2023). Furthermore, the intake of DDGS has been shown to significantly increase the relative abundance of several key probiotics in the gut. Notably, the phyla Firmicutes and Bacteroidota, which have been extensively studied, have been shown to possess probiotic properties. These microbial groups are closely associated with energy harvesting and immune system modulation, playing crucial roles in maintaining overall gut health (Sha et al., 2024). Notably, DDGS supplementation exerted significant antimicrobial selection pressure, suppressing Proteobacteria colonization compared to conventional diets. This phylogenetically heterogeneous phylum, encompassing enteropathogenic genera *Escherichia*, *Salmonella*, and *Proteus*, demonstrates particular sensitivity to DDGS-mediated microbiome remodeling, likely through β-defensin induction and bile acid-mediated pathogen inhibition (Latorre et al., 2018). Furthermore, dietary supplementation with DDGS was found to markedly enhance the relative abundance of core probiotic genera within the intestinal microbiota, with particularly pronounced effects observed for *Lactobacillus pontis* and *Romboutsia ilealis*. These beneficial bacterial strains, well-documented in scientific literature for their probiotic properties, demonstrate significant functional associations with enhanced gastrointestinal health. Their metabolic activities contribute to improved enzymatic digestion and optimized nutrient assimilation, while simultaneously engaging in immunomodulatory functions that strengthen host defense mechanisms (Rodrigues et al., 2020). These findings collectively suggest that dietary DDGS inclusion may induce beneficial modulatory effects on the gut microbiota ecosystem of Haidong chickens. Our hypothesis posits that DDGS supplementation could selectively enrich microbial taxa associated with energetic homeostasis and immunocompetence, potentially through multiple synergistic pathways (Rashid et al., 2021). Specifically, the phytoactive components in DDGS might enhance microbial-endocrine crosstalk to stimulate anabolic processes while modulating inflammatory responses, thereby enhancing growth performance parameters and improving egg quality parameters. This microbial reprogramming mechanism could fundamentally improve nutrient partitioning efficiency, directing metabolic resources toward both somatic development and reproductive biosynthetic pathways.

## Conclusion

**Figure 6:**
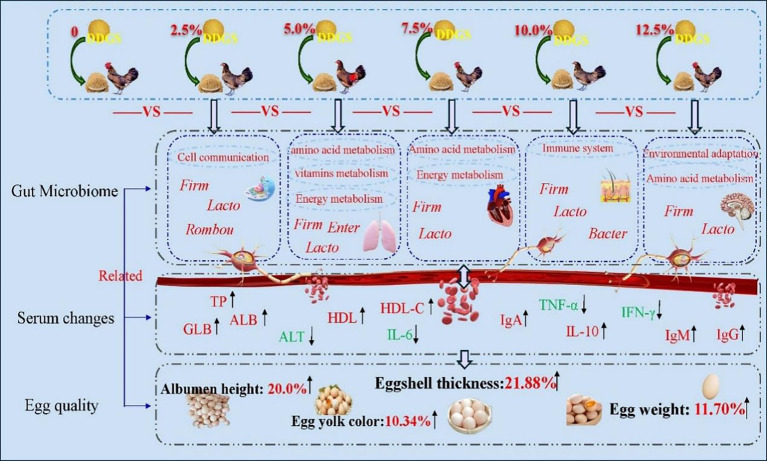
Graphical summary of the dietary supplementation with varying levels of DDGS improved egg quality by modulating gut microbial metabolism and regulating serum biochemical indices. The red font represented upregulation of bacterial flora, serum biochemical profiles, and KEGG function, while the green font represented downregulation. Firm, Firmicutes; Lacto, *Lactobacillus pontis*; Rombou, *Romboutsia ilealis*; Enter, *Enterococcus cecorum*; Bacter, *Bacteroides eaecicola*.

DDGS Supplementation in Haidong Chickens could improve serum physiological and biochemical properties during the egg-laying period, thereby improving egg quality. Some microbial communities related to intestinal immunity, food catabolism, and body growth and development were identified, such as *Lactobacillus pontis* and *Romboutsia ilealis* (Figure 6), which increase energy metabolism, amino acid metabolism, and the immune system, potentially improving the egg quality. Our findings provided a reference for the application of DDGS in animal diets.

## Data Availability

The datasets presented in this study can be found in online repositories. The names of the repository/repositories and accession number(s) can be found at: NCBI [accession: PRJNA1357349].
